# Sulforaphane in Cutaneous Disorders and Skin Injury: Mechanisms, Evidence, and Clinical Perspectives

**DOI:** 10.3390/nu18091444

**Published:** 2026-04-30

**Authors:** Hua Liu, Claire Y. Shi, Jed W. Fahey

**Affiliations:** 1 Stanley Division of Developmental Neurovirology, Department of Pediatrics, The Johns Hopkins University School of Medicine, Baltimore, MD 21287, USA; 2 Department of Medicine, The Johns Hopkins University School of Medicine, Baltimore, MD 21205, USA; jfahey@jhmi.edu; 3 Department of Psychiatry & Behavioral Sciences, The Johns Hopkins University School of Medicine, Baltimore, MD 21205, USA; 4 Department of Physiology, Pharmacology & Therapeutics, The Johns Hopkins University School of Medicine, Baltimore, MD 21205, USA; 5 iMIND Institute, The Johns Hopkins University School of Medicine, Baltimore, MD 21205, USA; 6 Institute of Medicine, University of Maine, Orono, ME 04469, USA

**Keywords:** atopic dermatitis, dysbiosis, immune response, inflammation, keratin disorders, Nrf2, phytochemical, psoriasis, skin aging, sulforaphane

## Abstract

Cutaneous disorders such as atopic dermatitis, psoriasis, acne vulgaris, and rosacea, together with UV-induced skin injury and photoaging, are highly prevalent conditions that involve varying contributions from dysregulated immune responses, cutaneous inflammation, oxidative stress, barrier dysfunction, microbiome alteration, and exogenous injury. However, these conditions are biologically heterogeneous and should not be regarded as a single mechanistic class. Sulforaphane, a naturally occurring isothiocyanate found primarily in broccoli and other cruciferous vegetables, has attracted interest in dermatology because of its antioxidant, cytoprotective, and context-dependent anti-inflammatory properties. Sulforaphane exerts its biological effects by modulating key signaling pathways, particularly the Keap1/Nrf2 pathway and, in some settings, NF-κB-related signaling, thereby reducing oxidative stress and inflammation, regulating immune responses, enhancing skin barrier function, and potentially influencing the cutaneous microbiome. Preclinical studies and limited human data suggest that sulforaphane may reduce erythema, edema, and other markers of cutaneous damage in selected settings. This comprehensive review explores the role of sulforaphane across heterogeneous cutaneous conditions, with emphasis on molecular mechanisms, disease-specific differences, current evidence, and discusses key translational constraints including formulation, delivery, lack of standardized dosing, and the limitations of cell culture and animal models for predicting human efficacy. Overall, sulforaphane should presently be regarded as a promising but still early-stage translational candidate in dermatology. Robust human efficacy data remain lacking for chronic inflammatory dermatoses such as psoriasis, atopic dermatitis, acne, and rosacea, whereas the strongest current human evidence relates to UV-associated skin outcomes and photoprotection.

## 1. Introduction

Cutaneous disorders and skin injury are among the most common chronic conditions, imposing significant physical, psychological, and socioeconomic burdens, warranting a multidimensional management approach. Notable disorders in this category include psoriasis, atopic dermatitis (AD, eczema), rosacea, and acne vulgaris (acne), as well as UV-induced skin injury such as sunburn. The prevalence of these conditions varies, with psoriasis affecting approximately 2–3% of the population, while AD impacts 15–20% of children globally and can persist into adulthood in many cases [1,2,3]. Rosacea affects around 5% of adults [4]. Acne, as one of the most common skin conditions, is prevalent among 85–90% of adolescents and young adults, and sunburn is almost universally experienced, with acute and long-term consequences including an increased risk of cutaneous malignancy [5,6,7,8,9,10].

While the mechanisms underlying these conditions are intricate, involving a combination of genetic predisposition, environmental factors, and immune system dysregulation [11,12], inflammation is a key feature in the pathogenesis of most of them [1,2,4,5,13], but these disorders should not be regarded as a single mechanistic class. Inflammatory responses are initiated through activation of the innate immune system in response to environmental insults, microbial infection, or genetic predisposition. Then, released inflammatory cytokines activate adaptive immunity, followed by keratinocyte dysfunction, oxidative stress, and compromised skin barrier integrity, leading to symptoms such as redness (erythema), swelling (edema), itching (pruritus), and sometimes pain [14,15,16]. However, the relative contribution of these processes differs substantially among diseases.

Specific mechanisms vary by disease. In conditions like psoriasis, an overreactive immune system triggers the production of pro-inflammatory cytokines such as TNF-α and IL-17, and results in the abnormal proliferation of skin cells, leading to the formation of thick, scaly plaques [17]. In AD, skin barrier dysfunction allows allergens and irritants to penetrate more easily, triggering immune activation, chronic inflammation and itching [18]. In acne, current evidence supports a more complex pathogenesis involving sebaceous gland biology, follicular hyperkeratinization, host–microbiome interactions, and dysbiosis, rather than simple bacterial overgrowth alone [5]. By contrast, sunburn is better understood as an acute UV-induced skin injury caused by exogenous damage and stress-response signaling, although inflammation remains an important downstream feature.

None of the current treatment options, including topical agents, systemic therapies, and biologics, have fully met the needs of affected patients, although effective for many of them [11,19,20,21,22,23,24,25,26,27]. Adverse effects, the need for consistent laboratory monitoring, and loss or lack of efficacy are common reasons for the decreased use of conventional therapies [28,29]. Consequently, many patients continue to face significant management challenges and persistent symptoms [30]. Treatment dissatisfaction presents a major barrier to achieving optimal care, and the most effective treatments are those that patients are willing to use consistently. Many affected individuals therefore seek additional therapeutic options. Thus, there is a need for more mechanism- and evidence-based, scientifically validated, and well-tolerated complementary or adjunctive medicine approaches for these conditions [31,32,33,34].

Much like other chronic diseases, these cutaneous disorders and injury states arise from perturbations in multiple signaling pathways, suggesting that targeting a single pathway is unlikely to yield effective prevention or treatment [35]. Therefore, there is a growing interest in exploring multi-targeted, cost-effective, readily available and non-toxic agents, that can complement conventional treatments [36]. Phytochemicals, small molecule analogs, and well-characterized simple plant extracts, have long been recognized for their diverse biological effects [37], making strategies that utilize multi-functional phytochemicals, or even the edible plants from which those compounds are isolated, particularly appealing.

**Figure 1:**
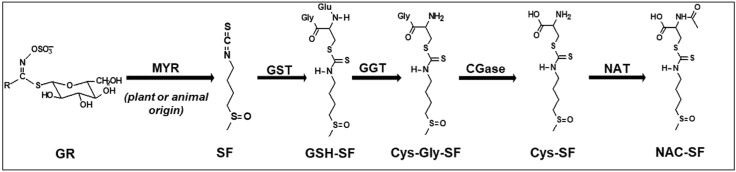
Conversion of glucoraphanin (GR) to sulforaphane (SF) and its metabolites glutathione-SF (GSH-SF), cysteinyl-glycine-SF (Cys-Gly-SF), cysteinyl-SF (Cys-SF), and N-acetyl-cysteinyl-SF (NAC-SF). The enzymes involved are, respectively, myrosinase (MYR), glutathione S-transferase(s) (GST), γ-glutamyl-transpeptidase (GGT), cysteinyl-glycinease (CGase), and N-acetyltransferase (NAT).

Sulforaphane [1-isothiocyanato-4-(methylsulfinyl)-butane; SF] is a naturally occurring isothiocyanate (ITC) derived from its biological precursor glucoraphanin (GR), a glucosinolate abundant in the cruciferous vegetable broccoli, and especially in broccoli sprouts and seeds [38,39,40,41]. Depending on its intended use, GR/SF can be classified as a food, dietary supplement, or drug [37,42,43]. SF is formed through the enzymatic hydrolysis of GR by the enzyme myrosinase during tissue disruption, processes like chopping or chewing activate this enzymatic conversion [38,41], and by the action of gastrointestinal microbiota [44,45]. Following absorption, SF rapidly forms glutathione conjugates, which facilitates its metabolism through the mercapturic acid pathway prior to excretion in the urine (Figure 1) [38]. This metabolic transformation is crucial as it influences the effective concentration of SF available for biological activity [46,47].

SF is a multi-functional phytochemical, with demonstrated antioxidant, anti-inflammatory, and anti-proliferative properties [48], along with anti-angiogenic effects [49,50,51]. Over the past three decades, a growing body of evidence, from in vitro studies to animal models and various clinical investigations, has highlighted SF’s remarkable ability to activate endogenous cytoprotective mechanisms, regulate redox balance, and inhibit pathogenic inflammatory signaling [48,52,53,54,55,56,57,58]. The pharmacologic versatility and favorable safety profile of SF position it as a viable candidate for safe, sustainable adjunctive therapy in managing these chronic skin conditions. However, the current evidence base remains weighted toward preclinical studies, and several translational issues, including model relevance, tissue exposure, formulation stability, and dose optimization, remain to be resolved. Even so, the available data support SF as a biologically active and translationally relevant candidate in dermatology, although its use should be evaluated based upon the evidence for each condition.

This review delves into the molecular mechanisms through which SF exerts its effects on heterogeneous cutaneous conditions and skin injury states and discusses the clinical evidence supporting its dermatologic applications. We also explore the challenges and future prospects for developing SF-based therapies for these conditions. The aim of this review is to provide a comprehensive overview of SF’s potential as a therapeutic agent in managing these dermatologic contexts, including its mechanisms of action, relevant preclinical and clinical studies, and the implications of its use in therapeutic regimens, while emphasizing disease-specific differences and the necessity for further research to clarify its benefits and applications in dermatology.

## 2. SF and Skin Health: Mechanisms of Action

The biologic effects of SF are rooted in its direct and indirect interactions with redox-sensitive pathways and inflammatory signaling systems that play critical roles in several cutaneous disorders and skin injury states. SF has been shown to modulate key stress-response and inflammatory pathways, inhibit the release of pro-inflammatory cytokines in selected experimental systems, and enhance the skin’s natural defense mechanisms [16,59]. Furthermore, its ability to activate the nuclear factor erythroid 2–related factor 2 (Nrf2) pathway, a critical regulator of cellular stress responses, underscores its potential relevance in mitigating oxidative stress and inflammatory amplification in the skin [60,61,62].

**Figure 2:**
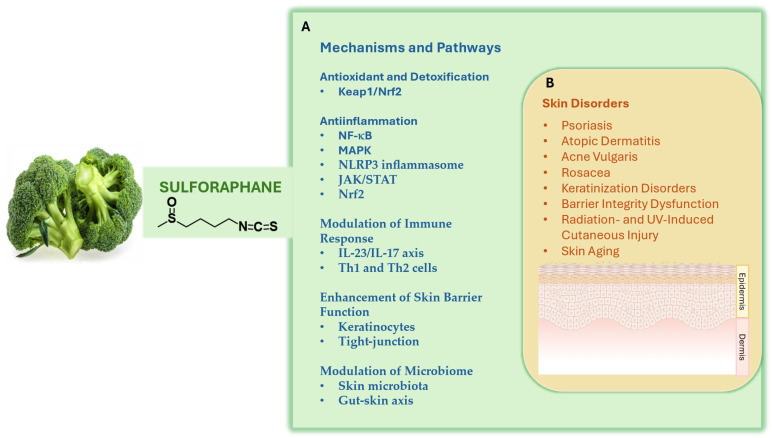
Sulforaphane and skin health. Sulforaphane has been shown in in vitro, preclinical, and clinical studies to engage multiple molecular and cellular pathways relevant to skin health (panel (**A**)), including activation of the Nrf2 antioxidant and phase 2 detoxification response; suppression of pro-inflammatory signaling (e.g., NF-κB, MAPK, and the NLRP3 inflammasome); modulation of immune function; preservation of barrier integrity via effects on tight-junction and keratinocyte homeostasis; and modification of the skin microbiome either directly or through the gut–skin axis. These mechanisms of action in panel (**A**) of the diagram are linked to beneficial effects on a range of skin disorders and conditions shown in panel (**B**), including inflammatory diseases (psoriasis, atopic dermatitis, acne vulgaris, and rosacea), disorders of keratinization and barrier dysfunction, UV-induced skin damage and photoaging, with specific pathways in panel A connecting to improvements in the listed conditions (panel (**B**)) through mechanistic studies, biomarker changes, and preclinical or clinical outcome measures.

SF mediates its biologic effects through various interconnected mechanisms primarily involving the modulation of antioxidant pathways, anti-inflammatory responses, and skin cell functions [63,64,65]. However, the relative importance of these mechanisms likely depends on disease context, route of delivery, tissue exposure, and duration of treatment. The key pathways and cellular interactions through which SF may exert these effects are outlined in Figure 2.

### 2.1. Antioxidant and Detoxification Pathways

Oxidative stress significantly contributes to the pathogenesis of various cutaneous disorders and skin injury states by driving an excess of reactive oxygen species (ROS) relative to antioxidant defenses in the skin. As the body’s largest organ, the skin serves as a protective barrier and is frequently exposed to environmental insults such as ultraviolet radiation (UVR), pollution, and pathogens, all of which can elevate oxidative stress [66,67,68]. Increased ROS levels can cause cellular damage, exacerbate inflammation, and alter immune responses, leading to conditions like psoriasis and AD. In other settings, such as UV-induced skin injury, oxidative stress may arise more directly from exogenous damage. In these diseases, oxidative stress not only aggravates inflammation but also contributes to the persistence of chronic lesions by promoting the secretion of pro-inflammatory cytokines and activating immune cells. Moreover, it can impair skin barrier function, further perpetuating inflammation and creating a cycle of tissue damage and immune activation. Thus, targeting oxidative stress pathways represents an important mechanistic strategy in several dermatologic contexts, although its relevance is likely to differ by disease.

One of the primary responses for cellular defense against oxidative and electrophilic stress is via the Nrf2 redox-sensitive transcription factor [69]. Under normal conditions, Nrf2 is sequestered in the cytoplasm by Kelch-like ECH associating protein 1 (Keap1), which facilitates its ubiquitination and proteasomal degradation. Upon exposure to oxidative stress or electrophilic compounds like SF, modifications of critical cysteine residues on Keap1 result in the release and nuclear translocation of Nrf2. Nrf2 then binds to antioxidant response elements (AREs) in promoters of target genes, enhancing the transcription of detoxifying and antioxidant enzymes such as heme oxygenase-1 (HO-1) and NAD(P)H quinone oxidoreductase 1 (NQO1), as well as enzymes involved in glutathione biosynthesis [70]. This transcriptional response enhances cellular antioxidant capacity, thereby reducing oxidative damage, which may be particularly relevant in cutaneous settings where redox imbalance contributes materially to tissue injury or inflammatory amplification.

SF is among the most potent naturally occurring inducers of mammalian cytoprotective enzymes through the Keap1/Nrf2/ARE signaling pathway [38]. Activation of Nrf2 has direct relevance to skin disease because it plays a significant role in counteracting oxidative stress, a major contributor to various inflammatory disorders [71,72,73,74,75]. As a master regulator of cellular redox homeostasis, Nrf2 is especially important for the skin, which is continuously exposed to environmental challenges [68,76,77]. Nrf2 also exhibits robust anti-inflammatory activity through several mechanisms, including modulation of redox metabolism, crosstalk with NF-κB, and direct inhibition of pro-inflammatory gene expression [78]. Additionally, Nrf2 may promote keratinocyte differentiation and regulate skin homeostasis [79,80]. Therefore, Nrf2 represents a biologically important and potentially targetable pathway in dermatologic disease [81,82]. At the same time, the consequences of Nrf2 activation are context dependent, and its role should not be assumed to be uniformly beneficial across all skin conditions. Notably, dimethyl fumarate (DMF), the only direct Nrf2 activator approved by both the European Medicines Agency and the US Food and Drug Administration, is used clinically to treat psoriasis and relapsing-remitting multiple sclerosis [83,84,85].

### 2.2. Anti-Inflammatory Mechanisms

Several cutaneous inflammatory disorders, including psoriasis and AD, are characterized by excessive production of pro-inflammatory cytokines and chemokines that contribute to disease pathophysiology, although the dominant inflammatory programs differ across diseases. SF exhibits potent anti-inflammatory properties by modulating various inflammatory pathways [86].

The nuclear factor kappa-light-chain-enhancer of activated B cells (NF-κB) is central to the inflammatory process, regulating the transcription of genes encoding cytokines (e.g., TNF-α, IL-1β, IL-6), adhesion molecules, inducible nitric oxide synthase (iNOS), and cyclooxygenase-2 (COX-2). In the context of inflammatory skin disorders, persistent NF-κB activation leads to production of these inflammatory mediators that contribute to keratinocyte hyperproliferation, immune cell recruitment, and cytokine-driven exacerbation of disease symptoms [87,88,89].

SF directly suppresses NF-κB signaling by preventing degradation of its inhibitor IκB-α, thereby blocking NF-κB nuclear translocation and limiting its transcriptional activity [86,90,91]. Furthermore, SF can indirectly inhibit NF-κB activation through crosstalk between Nrf2 and NF-κB [86,92]. In activated keratinocytes and dermal fibroblasts, SF inhibits the phosphorylation and nuclear translocation of NF-κB, leading to reduced transcription of inflammatory mediators like TNF-α, IL-6, IL-8, IL-1β, IL-17, and COX-2 [93,94,95]. These effects correspond with reduced skin inflammation and improved barrier function in experimental systems.

In imiquimod (IMQ)-induced psoriasis-like models, treatment with SF reduced nuclear translocation of NF-κB p65 and significantly decreased the expression of downstream inflammatory mediators [95]. This suppression of NF-κB correlated with an improvement in skin pathology and a decrease in keratinocyte proliferation, although such findings should be interpreted cautiously because IMQ-induced inflammation does not fully recapitulate human psoriasis.

Mitogen-activated protein kinases (MAPKs) are a family of protein kinases that also regulate the production of cytokines and mediators of inflammation, which involves regulating skin cell responses to stress and inflammatory stimuli. This family comprises three main groups: extracellular signal-regulated kinases (ERK), p38 MAPK, and c-Jun NH2-terminal kinases (JNK). Activation of these pathways leads to the release of proinflammatory cytokines such as IL-6, IL-8, and TNF-α, which can initiate inflammatory responses [96]. SF can inhibit the phosphorylation of key MAPK proteins, such as ERK, JNK, and p38 MAPK, thereby reducing inflammatory cytokine production and attenuating inflammatory responses [93,97,98,99,100].

Inflammatory activation can also occur through the nucleotide-binding domain leucine-rich repeat-containing family, pyrin domain-containing 3 (NLRP3) inflammasome, a protein complex involved in the production of pro-inflammatory cytokines such as IL-1b and IL-18, which contribute to disease progression and symptom severity. In vitro and in vivo studies have demonstrated that NLRP3 directly promotes keratinocyte proliferation and inflammatory cytokine expression [101], and circulating levels of these cytokines are significantly elevated in untreated psoriasis patients, further supporting their role in disease severity and systemic manifestations [102]. Studies have also confirmed a key role of NLRP3 in the inflammatory pathogenesis of acne, both in vitro and in vivo [103,104,105]. Recent research has also highlighted the significant role of the NLRP3 inflammasome in the development of AD [106,107]. SF has been shown to protect against pancreatic acinar cell injury by modulating Nrf2-mediated oxidative stress markers and suppressing activation of the NLRP3 inflammatory pathway [108]. Additionally, SF has been shown to suppress NLRP3 inflammasome activation through inhibition of NF-κB nuclear translocation and modulation of Nrf2-mediated miRNAs expression in murine microglia [109]. Notably, in a mouse model, SF suppresses the oxidative stress by upregulation of Nrf2, which subsequently inhibits activation of the NLRP3 inflammasome and DNA damage, thereby preventing and alleviating radiation-induced skin injury [110]. 

SF also modulates Janus kinase/signal transducer and activator of transcription (JAK/STAT) signaling, further attenuating the inflammatory cascade [95,97,99,100,111,112]. The convergence of these signaling networks contributes to the inflammatory activation in several acute and chronic cutaneous conditions, but the extent to which SF can meaningfully modulate these pathways in human disease remains uncertain and is likely to vary across dermatologic contexts.

### 2.3. Modulation of Immune and Cellular Responses

Immune responses and several inflammatory skin disorders are closely linked, with conditions like psoriasis and AD arising from dysregulated immunity where genetic and epigenetic alterations, metabolic changes, environmental factors, and microbiome dysbiosis trigger abnormal activation of immune cells (e.g., T-cells, macrophages) and excessive production of cytokines, leading to chronic inflammation, itching, and skin barrier disruption [113]. In these disorders, the immune responses may be excessive, prolonged, or inappropriate, contributing to the development and persistence of disease symptoms, although the dominant immune programs differ substantially across diseases.

Specific immune pathways play crucial roles in the pathogenesis of common inflammatory dermatoses. For instance, psoriasis is characterized by Th17 cell involvement [114,115,116], which is a major driver of inflammation, and AD is primarily linked to an imbalance between Th2 cells and Th1 cells [2,117,118,119].

SF has been reported to exert immunomodulatory effects through interacting with various immune cell types, including T cells and macrophages, thereby modulating their activity [120,121,122,123]. For example, in psoriasis experimental studies SF may inhibit Th17 cell activation. By influencing immune-cell activation and the secretion of inflammatory mediators, SF can help reduce the inflammatory environment that exacerbates skin diseases [122]. These findings provide clear mechanistic support for immunomodulatory activity of SF, although their translation into clinically meaningful benefit in human dermatologic disease remains to be demonstrated.

### 2.4. Effects on Skin Barrier Function

Compromised skin barrier function plays a pivotal role in the pathophysiology of several cutaneous disorders, particularly AD, and may also contribute to psoriasis, acne, and rosacea. Inflammatory cytokines can disrupt the integrity of the skin’s protective barrier, and a weakened physical and chemical barrier permits allergens and pathogens to infiltrate the skin, triggering immune responses and contributing to chronic inflammation [124,125,126,127,128,129,130,131,132].

Keratinocytes, the predominant cell type in the epidermis, are essential for maintaining skin homeostasis and orchestrating the inflammatory response. SF has been shown to enhance the viability of keratinocytes under oxidative stress conditions [93,133]. By inducing the Nrf2-mediated production of antioxidant enzymes, SF helps to sustain keratinocyte integrity, thereby potentially supporting skin barrier function.

By way of Nrf2 activation, SF may enhance the expression and stability of key barrier proteins, such as filaggrin, loricrin, and involucrin, within keratinocytes [134]. Additionally, Nrf2-mediated improvement of antioxidant status reduces peroxidation of membrane lipids, preserving the architecture of the skin barrier [95,112,135]. These findings suggest that SF may, in some settings, contribute to the restoration of epidermal integrity.

SF also influences keratinocyte differentiation, promoting a more balanced state that supports normal skin structure [95]. This modulation may aid in restoring proper barrier function and strengthening the skin’s protective layer.

Although direct evidence remains limited in this organ (the skin), tight-junction preservation may represent an additional mechanism by which SF supports epidermal barrier function in inflammatory dermatoses. Evidence from other (non-cutaneous) barrier tissues indicates that SF can oppose junction disruption during inflammatory and oxidative stress, including Nrf2-dependent maintenance of blood–brain barrier tight-junction proteins and function [136], protection against NSAID-related intestinal barrier injury [137], reduction in lipopolysaccharide-induced epithelial permeability [138], and increased intestinal tight-junction protein expression in vivo [139]. Although these data do not yet establish a direct cutaneous tight-junction effect, they provide a compelling rationale for considering tight-junction preservation as one possible component of the barrier-related activity of SF in selected dermatologic contexts.

### 2.5. Potential Effects on the Microbiome

Recent research highlights the crucial role of human microbiota, encompassing gut and skin communities, in both physiological and pathological processes. These microbial populations are essential for maintaining homeostasis, modulating immune response, and defending against pathogens. Dysbiosis, characterized by imbalances in these communities, can impair barrier functions, disrupt inflammatory and immune responses, and potentially initiate disease development [140,141].

Skin microbiota, a complex ecosystem of microorganisms, is vital for skin integrity. Dysbiosis impairs the skin barrier, increasing water loss and allowing harmful microbes and toxins to enter, triggering inflammation. Specific microbial shifts have been documented in various skin conditions. For example, AD is associated with decreased microbial diversity and an overgrowth of pathogenic *Staphylococcus aureus* [142,143]. In psoriasis, although there is no convincing conclusion regarding whether the diversity of the microbial community on psoriatic lesional skin is lower than that on healthy skin, increased *S. aureus* abundance and decreased *S. epidermis* abundance have been observed in psoriatic lesions [144,145].

Meanwhile, an increasing body of evidence links disrupted gut flora to immunological and metabolic disorders manifesting in the skin, contributing to the onset of conditions such as AD, psoriasis, and rosacea [146,147,148,149,150]. Although the precise mechanisms connecting the gut and skin microbiomes remain inadequately understood [146,149,151], several microbial alterations have been reported. For instance, levels of *Clostridium difficile*, *Escherichia coli*, and *S. aureus* were higher in individuals with AD compared to the healthy controls [152,153]. Patients with psoriasis exhibit altered gut microbiomes with increased *Prevotella* spp. and decreased *Lachnospira* spp. and *Akkermansia muciniphila*, along with reduced microbial diversity compared to healthy individuals [154,155,156]. Rosacea has also been linked to significant microbial alterations [157], including an increased prevalence of *Helicobacter pylori* in moderate-to-severe cases [158,159]. This underscores the significance of the gut-skin axis as a potentially important pathway in the pathophysiology of these conditions [140]. Further investigation of this axis could illuminate how alterations in gut microbiota composition and diversity influence epidermal differentiation and metabolism, since dysbiosis can trigger immune responses, promote inflammation, and compromise skin barrier integrity [141,144,160,161,162].

Given the association between dysbiosis and several dermatologic conditions, addressing microbial imbalances presents a potential therapeutic strategy [140]. SF has emerged as a potent modulator of the gut microbiome, increasing beneficial bacteria such as *Bacteroidetes* spp., enhancing the production of anti-inflammatory metabolites like short-chain fatty acids (SCFAs), and improving gut barrier function [163,164,165,166,167,168,169,170,171,172]. Studies involving inflammatory bowel disease (IBD) demonstrate that SF supplementation restores microbial balance and reduces inflammation. In mouse models of colitis, SF improved gut barrier integrity and modulated the microbial composition, resulting in diminished inflammation [173,174,175]. In an autism spectrum disorders (ASD)-like rat model and ASD patients, SF treatment alleviated social deficits linked to the modulation of gut microbiota [176]. Additionally, oral SF administration ameliorated the progression of hyperuricemia by reprogramming the gut microbiome and metabolome in a rat model [177]. Therefore, by reducing pathogenic microbes and promoting a more diverse microbiome, SF can mitigate inflammation, metabolic disorders, and dysbiosis, ultimately improving systemic health and potentially impacting skin conditions.

While most microbiome research has concentrated on the gut, studies that directly examine SF’s effects on skin microbiota remain limited. Existing work has mainly explored how SF modulates skin inflammation, immune responses, and cellular health mechanisms that could indirectly alter microbial communities and which indicate potential dermatologic effect. The gut lining (alimentary canal) and the skin are the two principal sites where epithelial cells and their tight junction’s interface directly with extensive microbial ecosystems. Both allochthonous and autochthonous microbial communities occupy distinct niches across the skin’s surface, and, as with the gut, their composition is plausibly influenced by phytochemicals such as SF [178,179].

Overall, SF’s influence on both the gut and skin microbiomes supports interest in the possibility that microbiome-related effects may contribute to some of its biologic actions. However, direct evidence that SF improves dermatologic disease through microbiome modulation remains limited. Further exploration of the intricate connections between diet, microbiome modulation, and skin health is necessary to clarify the clinical relevance of SF in dermatologic disease.

In summary, through activation of Nrf2 signaling, inhibition of pro-inflammatory pathways such as NF-κB and MAPK, modulation of immune responses and keratinocyte function, and possible effects on the gut–skin axis, SF may influence several biologic processes relevant to dermatology. Understanding these mechanisms not only helps clarify where SF has mechanistic plausibility but also sets the stage for future studies exploring its clinical applications in dermatology.

## 3. Evidence from Specific Cutaneous Disorders and Skin Injury

Although substantial preclinical evidence supports biologic activity of SF in several dermatologic contexts, well-designed clinical trials demonstrating therapeutic efficacy remain lacking for many of the chronic conditions discussed below. Herein, we critically review the preclinical and emerging clinical evidence for SF in conditions such as psoriasis, AD, and other dermatologic disorders (Figure 2).

The cutaneous conditions discussed in this review should not be regarded as a single mechanistic class. Psoriasis and atopic dermatitis are immune-mediated inflammatory dermatoses, although they differ markedly in immune architecture [180,181], whereas UV-induced skin injury (sunburn), radiation dermatitis, and skin aging are driven more directly by exogenous damage, oxidative stress, and tissue remodeling. Acne and rosacea represent additional mechanistically distinct contexts involving pilosebaceous, innate immune, neurovascular, microbial, and barrier-related mechanisms, while keratinization disorders are centered more directly on structural and differentiation abnormalities of the epidermis. Accordingly, the most plausible point of convergence for SF is not one shared inflammatory mechanism, but partial overlap in oxidative stress handling, barrier stress, and Keap1/Nrf2-linked cytoprotective pathways.

### 3.1. In Vitro and Mechanistic Studies

Cultured human keratinocytes and dermal fibroblasts have been extensively employed in cell, co-culture, human skin equivalent, and ex vivo studies. In these studies, pretreatment with SF confers protection against oxidative damage from hydrogen peroxide, UVR, particulate matter (PM) exposure, and inflammatory cytokines [65,93,95,133,182,183,184]. These protective effects are abrogated when Nrf2 is silenced or knocked out, confirming the critical role of this pathway [76,133]. Furthermore, SF is shown to reduce the secretion of inflammatory chemokines (such as CCL17, CCL20, CCL22 and CXCL8) [184,185] and matrix metalloproteinases (MMPs) [65,186], while also normalizing abnormal keratinocyte proliferation observed in models of psoriasis and acne [60,187,188]. However, these experimental systems do not fully capture the complexity of human skin disease, and the concentrations used in vitro may not reflect achievable tissue exposure in vivo. Of note, Yehuda and colleagues demonstrated that SF reduced expression and secretion of key psoriasis-related pro-inflammatory cytokines in human monocytes, macrophage-like cells [94]. Taken together, these studies demonstrate that SF can modulate stress-response and inflammatory pathways in relevant cell systems and provide an important mechanistic basis for further translational investigation, although they are not in themselves evidence of clinical efficacy.

### 3.2. Psoriasis

Psoriasis is a common, chronic, proliferative, and immune-mediated dermatosis characterized by epidermal hyperplasia, scaling, erythema, and infiltration of activated immune cells [1,116,189,190,191]. It is no longer thought of as a disorder that affects only the skin, but is instead seen as a systemic inflammatory disorder associated with an increased prevalence of comorbid conditions, including psoriatic arthritis, cancer, depression, metabolic syndrome, type 2 diabetes, and cardiovascular disorders [17,192,193,194,195]. These complications contribute to compromised quality of life, reduced work productivity, increased physical disability, and can even lead to impaired social functioning [196].

**Preclinical Studies:** The pathophysiology of psoriasis is complex and dynamic. Dysregulation of the IL-23/IL-17 axis, oxidative stress, and NF-κB activation are key features driving disease pathogenesis. The IMQ-induced murine model is widely used as a psoriasis-like inflammatory model, but it does not fully recapitulate the clinical and immunologic complexity of human [197,198]. Two studies have evaluated SF in this model, in which the efficacy of SF was not attributable to a single molecular target, but rather to modulation of multiple pathways relevant to psoriasis-like inflammation in this experimental setting [95,199].

In one of these studies, Du et al. (2022) observed that intraperitoneal (i.p.) administration of SF ameliorated IMQ-induced skin lesions [199]. Mechanistically, SF upregulated antioxidant genes including peroxiredoxin 1 (Prdx1; an antioxidant enzyme that catalyzes the reduction of hydrogen peroxide), NQO1, HO-1, and glutathione synthetase (Gss), while reducing lipid peroxidation (TBARS/MDA) in IMQ-stimulated mouse skin [199]. Meanwhile, flow cytometry revealed fewer Th1 and Th17 cells in draining lymph nodes and spleen, consistent with attenuation of IL-23/IL-17 axis signaling. Additionally, docking studies suggested a potential interaction between SF and RORγt (the master transcription factor for cytokine IL-17), although this finding remains inferential.

Using the same model, Ma et al. (2023) reported that systemic i.p. SF significantly reduced skin thickness, scaling, and erythema [95]. These clinical improvements were accompanied by decreased epidermal hyperplasia and dermal inflammatory cell infiltration, as well as reduced expression of psoriasis-associated stress keratins (K16 and K17) and the proliferation marker Ki67, indicating normalization of keratinocyte hyperproliferation [95]. Furthermore, SF restored diminished Nrf2 expression in psoriatic lesions, increased expression of canonical antioxidant genes, and reduced STAT3 and NF-κB activation in skin tissue. These changes correlated with lower levels of IL-1β, IL-6, and CCL2 in both lesional skin and in IL-22/TNF-α-stimulated HaCaT keratinocytes treated with SF.

Taken together, these preclinical results suggest that SF can attenuate psoriasis-like pathology in experimental systems through restoration of Nrf2-mediated antioxidant defenses, suppression of NF-κB and STAT3 signaling, as well as reduction in pro-inflammatory mediators and Th1/Th17-associated responses. Although these findings should be interpreted as mechanistic and preclinical evidence rather than as proof of clinical efficacy in psoriasis, they support further investigation of SF as a potential therapeutic strategy in psoriasis.

**Clinical evidence:** Human data supporting SF in psoriasis remain limited. Earlier work by Yehuda et al. (2012) reported that ITCs modulate psoriasis-relevant inflammatory mediators in human monocytes and macrophage-like cells, and additionally demonstrated suppression of psoriasis-associated cytokine expression and secretion in ex vivo cultures of inflamed human skin using 4-methylthiobutyl isothiocyanate (the reduced form of SF) rather than SF [94]. While these findings provide preliminary human-cell support for SF activity and human-tissue support for closely related ITC-mediated modulation of psoriasis-relevant pathways, they do not include clinical outcome measures. Well-designed clinical trials demonstrating therapeutic efficacy of SF in psoriasis are still lacking, and further translational research is needed.

### 3.3. Atopic Dermatitis (AD, Eczema)

AD is a chronic relapsing inflammatory dermatosis characterized by severe skin lesions such as pruritus, erythema, rash, edema, dryness, and skin hypersensitivity [3]. Because of these features, AD patients often suffer from sleep deprivation, anxiety, and stress, which can lower their quality of life. Individuals with AD are also at increased risk of developing other chronic inflammatory conditions, including asthma and allergic reactions [13,200].

AD has a complex etiology involving genetic, immunologic, and environmental factors. A central feature of AD pathogenesis is skin barrier dysfunction, which promotes enhanced penetration of allergens, irritants, and microbes and thereby facilitates ongoing immune activation [201,202]. AD is often accompanied by elevated serum immunoglobulin E (IgE) levels [27]. Skin microbiome abnormalities and oxidative stress are also key components of pathogenesis in AD [13,25,142]. Genetic and acquired barrier abnormalities, including filaggrin loss-of-function mutations, defects within the epidermal differentiation complex, altered stratum corneum lipid composition with shortened ceramide and fatty acid chains, and tight junction disruption, compromise epidermal integrity. These changes lead to increased transepidermal water loss, elevated skin pH, and enhanced penetration of allergens and microbes [200,201,202]. In contrast to psoriasis, which is driven predominantly by IL-23 and Th17-centered immunity, AD is characterized mainly by type 2 immune polarization. Key roles are played by cytokines such as IL-4 and IL-13, which further impair barrier function and amplify inflammation. Chronic lesions acquire additional Th22, Th17, and Th1 components (e.g., IL-22, IL-17A, IFN-γ), while pruritogenic cytokines such as IL-31, IL-4, IL-13, and TSLP act directly on sensory neurons to promote itch, scratching, and mechanical barrier damage. This establishes a self-reinforcing cycle linking barrier disruption, type 2-skewed inflammation, and pruritus [13,203].

Although current therapies have improved outcomes for many patients, interest remains in adjunctive or complementary approaches that might modulate oxidative stress, inflammatory amplification, or barrier-related dysfunction without displacing established disease-targeted treatments [27].

**Preclinical Studies:** Two studies have examined SF in 2,4-dinitrochlorobenzene (DNCB)-induced AD-like dermatitis models in mice using i.p. or subcutaneous (s.c.) administration. SF pre- or post-treatment attenuated scratching behavior, suppressed skin thickening and spongiosis, and reduced serum IgE levels, consistent with anti-inflammatory and barrier-restorative actions [112,204].

Wu et al. (2019) reported that SF administered i.p. three times weekly reduced ear thickness, dermatitis scores, and scratching behavior in the AD mouse model [112]. Histologically, SF decreased eosinophil and mast cell infiltration, while significantly lowering serum IgE levels [112]. The study also demonstrated increased Nrf2 and HO-1 expression in lesional skin following SF treatment, along with reduced phosphorylation of JAK2 and STAT3, and decreased IL-6, IL-1β, and TNF-α levels [112].

Using s.c. SF administration after AD establishment in the DNCB-induced AD-like mouse model, Alyoussef observed attenuated clinical lesions and reduced expression of NF-κB, TNF-α, IL-1β, IL-4, IgE, and caspase-3 in skin tissue. These changes were accompanied by increased Nrf2 and IL-10 expression and reduced oxidative DNA damage [204].

Taken together, these independent preclinical studies suggest that systemic SF treatment can ameliorate AD-like features (redness, itching, swelling) in experimental models by inhibiting inflammation (NF-κB, Th2-relevant chemokines, and pro-inflammatory cytokines), enhancing antioxidant defenses via Nrf2 activation, and lowering immune-associated markers such as eosinophils, mast cells, and IgE. Although these findings derive from chemically induced murine dermatitis models and do not establish clinical efficacy in human AD, they provide meaningful preclinical support for the biologic potential of SF in this setting. However, well-designed human clinical trials evaluating SF specifically in AD remain lacking. Further translational studies are needed to confirm efficacy, establish dosing parameters, and determine its clinical relevance as a possible adjunctive strategy.

### 3.4. Acne Vulgaris and Rosacea

At present, evidence for SF in acne and rosacea remains limited or indirect, and no well-designed clinical trials establish therapeutic efficacy in either condition. Acne vulgaris is a multifactorial disorder of the pilosebaceous unit and should not be treated as a straightforward counterpart to immune-mediated inflammatory dermatoses such as psoriasis or AD. Contemporary models emphasize the interplay among sebaceous gland activity, altered sebocyte differentiation, follicular hyperkeratinization, host–microbiome interactions, and dysbiosis within the pilosebaceous niche [205,206]. Rosacea is also mechanistically distinct from psoriasis and AD, involving dysregulated innate immunity, neurovascular signaling, barrier dysfunction, and microbial and environmental triggers [207]. However, the mechanistic rationale, including the roles of oxidative stress, NF-κB signaling, and dysregulated innate immunity in these conditions, suggests that SF may represent a candidate for future investigation, although this rationale is more indirect than in psoriasis, AD, or UV-related skin injury.

Most notably, sulforaphene, the structurally related ITC derived from radish seeds, has been shown to inhibit *Cutibacterium acnes* growth and reduce *C. acnes*-induced pro-inflammatory cytokine production in HaCaT keratinocytes and THP-1 monocytes through suppression of NF-κB signaling, including modulation of IL-1α [187]. Additionally, a small clinical study of a 6% broccoli stem extract cream demonstrated improvements in post-acne macular erythema and hyperpigmentation, attributed to combined antioxidant, anti-inflammatory, and anti-melanogenic effects of broccoli-derived phytochemicals, including SF [208]. However, this study focused on post-inflammatory sequelae rather than active inflammatory lesions, and the multicomponent formulation limits attribution specifically to SF.

Commercial nutraceutical formulations for rosacea have incorporated SF-containing extracts as one component among multiple antioxidants and bioflavonoids, often citing SF’s Nrf2 activation and NF-κB inhibition as putative mechanisms [209]. However, these claims are not supported by controlled, peer-reviewed clinical trials and the specific contribution of SF cannot be known.

Although the findings cited here are preliminary and involve either a different but functionally related ITC, sulforaphene [210], or multicomponent botanical formulations, they suggest that SF-related compounds may warrant further study in acne and rosacea. At the same time, acne and rosacea are mechanistically distinct from psoriasis and AD, and current evidence is insufficient to support clinical inferences for SF in either condition. Dedicated preclinical and early-phase clinical studies will be required to determine whether SF has any meaningful disease-specific role in these common disorders.

### 3.5. Keratinization Disorders and Barrier Integrity Dysfunction

A distinctive aspect of SF’s dermatologic profile is its capacity to reprogram keratin biosynthesis and compensate for structural defects in the keratin network, thereby potentially improving barrier integrity.

In an epidermolysis bullosa simplex (EBS) mouse model, we demonstrated that in utero treatment with SF (i.p. injections of SF to the pregnant mother) dramatically reduced blistering and improved skin integrity in newborn keratin-14-deficient mice by inducing expression of more resilient keratins, notably K16 and K17, thereby reinforcing the keratin cytoskeleton [16]. This study provided early proof-of-concept for SF as a potential disease-modifying agent in genetic skin fragility disorders. In mechanistic follow-up studies, our colleagues showed that SF induces K16 and K17 expression in mouse epidermis through both Nrf2-dependent and -independent pathways [211]. Nrf2 knockout and Keap1-hypomorphic mice established a requirement for Nrf2 in part of the keratin response and in phase 2 gene induction, while pharmacologic inhibition and genetic perturbation of MEK–ERK and AP-1 pathways revealed an Nrf2-independent contribution to K16 induction [211]. This work underscores that SF’s effects on keratin expression are not solely attributable to Nrf2 and may involve classical stress-responsive MAPK/AP-1 signaling. We ultimately conducted a clinical study in which we applied ca. 71 nmol/cm^2^ of SF in a SF-rich broccoli sprout extract (BSE), to the inner arm of human subjects daily for 7 days. This treatment activated Nrf2, up-regulated K17, had variable effects on K16 and K6 expression, and did not alter K14 or K5 expression in the treated skin [212].

In Keratin (Krt)16-null mice, which develop palmoplantar keratoderma (PPK)-like paw lesions, the same research group found that early defects in keratinocyte differentiation and downregulation of Krt9 precede lesion onset [213]. Topical SF prevented development of PPK-like lesions and restored Krt9 expression and terminal differentiation, suggesting that SF can normalize volar epidermal differentiation programs.

Schäfer et al. investigated the consequences of sustained Nrf2 activation in keratinocytes using keratinocyte-specific constitutively active Nrf2 transgenic mice and topical Nrf2 activators, including SF and tBHQ [67]. Chronic high-level Nrf2 activity produced epidermal thickening, hyperkeratosis, impaired desquamation, altered lipid metabolism, and a secondary inflammatory infiltrate resembling lamellar ichthyosis. Transcriptomic analysis identified multiple Nrf2-regulated barrier components, including cornified envelope proteins (Sprr2d/2h) and the serine protease inhibitor Slpi, implicating Nrf2 in the regulation of desquamation and lipid handling [67].

Taken together, these findings suggest that moderate, targeted Nrf2 activation by SF may be beneficial in keratin fragility disorders and PPK-like states. However, chronic or excessive Nrf2 activation can itself provoke barrier dysfunction and inflammation [82,214]. If SF or related approaches are to be used in keratinization disorders, the magnitude and duration of Nrf2 activation must therefore be finely tuned.

### 3.6. Radiation- and UV-Induced Cutaneous Injury

In addition to direct DNA damage, radiation and UV promote the generation of reactive oxygen intermediates that can cause oxidative damage, tissue remodeling, and inflammatory responses, ultimately leading to skin aging and tumor formation [215]. In the skin, Nrf2 activation in keratinocytes and fibroblasts leads to increased resilience against UV-induced oxidative damage, chemical insults, and biologically generated free radicals [68,76,82,216,217]. Animal studies have shown that Nrf2 deficiency increases susceptibility to cutaneous oxidative injury, while pharmacologic activation by SF can restore adaptive capacity and reduce lesion severity in selected experimental settings [95,112,133,199,204,214,218,219]. Protection against UVR-induced skin damage using SF or BSE has therefore been extensively investigated by us and others.

**Preclinical studies:** In a murine thigh X-ray irradiation model, Wei et al. showed that SF reduced dermal and hypodermal fibrous hyperplasia and histologic damage [110]. SF increased Nrf2 and downstream antioxidant gene expression, decreased ROS and markers of oxidative damage (4-HNE, 3-nitrotyrosine), and suppressed components of the NLRP3 inflammasome, including NLRP3, caspase-1, and IL-1β [110]. An earlier related report from the same group corroborated these protective effects [220]. These data position SF as a modulator of radiation-induced oxidative stress and inflammasome-driven inflammation in skin, suggesting possible utility in radiation dermatitis or as a protective adjunct to radiotherapy.

Chaiprasongsuk et al. examined SF in UVA-irradiated HaCaT keratinocytes and BALB/c mouse dorsal skin [186]. Repetitive UVA exposure increased MMP-1 expression and collagen degradation via activation of MAPK pathways (ERK, JNK, p38) and AP-1 signaling. Pretreatment with SF, via Nrf2 activation, reduced UVA-induced MMP-1 expression, preserved collagen integrity, and diminished MAPK and AP-1 phosphorylation [186]. Nrf2 knockdown abrogated these protective effects, confirming Nrf2 dependence.

Additionally, topical application of SF-containing BSE induced the phase 2 response in the epidermis of SKH-1 hairless mice, increasing the protein levels of NQO1, glutathione S-transferase A1, and HO-1, three representative phase 2 enzymes [221,222]. In an HR-1 hairless mouse model, SF oral administration significantly reduced UVB-induced skin thickness, COX-2 protein expression, and epidermal hyperplasia [93].

**Clinical studies:** In two randomized, double-blind, placebo-controlled independent clinical trials in healthy human subjects, our group observed protection against erythema induced by UVR exposure of human skin following topical application of SF from broccoli sprouts [62,135]. Furthermore, topical application of SF-containing BSE increased the enzyme activity of NQO1 in a dose-dependent manner in human skin punch biopsies, thus providing direct evidence for induction of the phase 2 response in humans [221].

In another study, Kleszczyński et al. investigated SF in ex vivo human full-thickness skin exposed to UVR, demonstrating protection against UVR-induced damage by reducing the number of sunburn cells, preventing the depletion of the antioxidant enzyme catalase, and protecting against apoptosis as shown by reduced caspase-3 activation via Nrf2-dependent induction of HO-1 and other antioxidant enzymes [182]. This work provides direct evidence that SF pre-treatment can activate Nrf2 in human skin and attenuate UVR-induced damage.

Most recently, we reported that in healthy human skin exposed to UVB, short-term oral GR administration significantly upregulated the cytoprotective/antioxidant enzyme NQO1 and reduced local pro-inflammatory mediators IL-1β, TNF-α, IL-6, IL-17, STING, and CYR61 in the skin, with confirmed delivery of SF to dermal tissue [223]. Although this small, short-duration study did not demonstrate a clear clinical photoprotective endpoint (e.g., reduced erythema), it confirmed measurable biological activity in human skin following oral, rather than topical administration.

Taken together, this is one of the strongest translational areas for SF in dermatology. Current human data support photoprotective effects and measurable biologic activity in skin, whereas its role in routine clinical management of radiation dermatitis remains undefined.

### 3.7. Skin Aging

While skin aging is not classified as an inflammatory skin disorder, it shares several pathophysiological mechanisms in which SF has demonstrated biologic activity. Therefore, SF has been investigated in the context of skin aging, particularly as a prototypical small-molecule activator of the Keap1/Nrf2 cytoprotective pathway with relevance to both intrinsic skin aging and photoaging [81,82]. Preclinical work consistently shows that SF attenuates oxidative stress, matrix degradation, and dermal structural decline, while early human studies demonstrate pharmacologic delivery to skin and modulation of UV-responsive pathways, albeit without yet proving macroscopic “anti-aging” efficacy.

**Preclinical studies:** In murine models of spontaneous aging, chronic dietary SF or GR supplementation improves multiple histological and molecular hallmarks of aged skin. Petković et al. observed robust up-regulation of Nrf2 and its downstream targets NQO1 and HO-1 in skin following a three-month SF-supplemented diet, accompanied by reduced ROS levels and lower MMP-9 protein abundance in aged animals [60]. Histologically, SF treatment improved collagen organization and dermal structure in both young and old mice, suggesting that long-term Nrf2 activation may help prevent and possibly partially reverse age-associated dermal extracellular matrix (ECM) deterioration.

Similar findings were reported in a senescence-accelerated mouse prone 1 (SAMP1) model. Chawalitpong et al. administered GR-enriched kale (metabolized in vivo to SF) to SAMP1 mice for 39 weeks and documented suppression of dorsal skin thinning, an overall reduction in composite senescence scores, and increased collagen production in skin [224]. Mechanistically, GR/SF intake enhanced nuclear translocation of Nrf2 and HO-1 expression, and activated TβRII/Smad3 signaling, implicating combined antioxidant and pro-collagenogenic actions. Together, these studies support the concept that chronic, low-dose SF exposure can mitigate intrinsic skin aging by decreasing oxidative stress and preserving dermal ECM.

A substantial body of preclinical literature also links SF-mediated Nrf2 activation to protection from UV-driven skin damage, an important contributor to clinical photoaging [77]. As described in Section 3.6, in a mouse model of UVA exposure, Chaiprasongsuk et al. demonstrated that Nrf2 knock-down or genetic deficiency augments UVA-induced MMP-1 expression and collagen loss via MAPK/AP-1 pathways, whereas topical SF activates Nrf2, suppresses MMP-1 up-regulation, restores type I collagen, and reduces UVA-induced epidermal thickening [186]. These data directly link SF-induced Nrf2 signaling to inhibition of the canonical collagen-degrading axis that underlies wrinkle formation.

UVB models reinforce this protective role. Saw and colleagues showed that Nrf2-null mice develop more sustained UVB-induced inflammation, more sunburn cells, higher IL-1β and IL-6 expression, and greater keratinocyte apoptosis than wild-type controls; topical SF markedly reduced sunburn cell formation and inflammatory markers in Nrf2-competent but not Nrf2-deficient mice, demonstrating Nrf2 dependence [225]. A subsequent study from the same group reported increased UVB-induced ECM degradation and pro-MMP-9 expression in Nrf2-knockout mice, further implicating Nrf2 in preservation of dermal connective tissue [226]. These findings strongly suggest that SF, through Nrf2 activation, can attenuate both inflammatory and structural components of UVB-driven photoaging.

Work from our group provided foundational evidence that SF-rich BSE induces phase 2 cytoprotective enzymes in murine skin and reduces UV-induced inflammation and edema [135]. More recently, Li and co-workers used integrated metabolomic, methylomic, and transcriptomic profiling in UVB-irradiated HaCaT keratinocytes to show that UVB drives extensive metabolic rewiring and epigenetic reprogramming in pathways related to oxidative stress, TGF-β signaling, and matrix regulation. Co-treatment with SF attenuated many of these UVB-induced changes, supporting a role for SF in limiting early molecular events that can contribute to photoaging and photo-carcinogenesis [64].

In the context of air pollution-related skin aging, SF has been shown to counteract fine particulate matter (PM2.5)-induced oxidative and paracrine damage in cutaneous cells. Using keratinocyte-melanocyte and keratinocyte-fibroblast co-culture systems exposed to diesel PM2.5, Ko et al. reported that SF markedly reduced intracellular ROS in keratinocytes and suppressed NF-κB-dependent expression of pro-inflammatory cytokines (TNF-α, IL-1β, IL-6, COX-2), melanogenic mediators (endothelin-1, PGE2), and ECM-degrading or pro-senescent factors (CCN1, MMP-1), while preserving type I procollagen in fibroblasts and attenuating melanogenesis in melanocytes [65]. These data support a model in which SF, via Nrf2 activation and NF-κB inhibition, dampens PM2.5-triggered oxidative stress in keratinocytes and normalizes their paracrine signaling to neighboring pigment cells and fibroblasts, thereby mitigating pollution-induced dyspigmentation and collagen breakdown. A recent review highlights this work as a key example of how broccoli-derived ITCs can modulate PM2.5-induced skin inflammation, barrier dysfunction, and premature aging phenotypes, positioning SF as a promising anti-pollution agent for skin-care and dermatologic applications [227].

**Clinical studies:** Human data on SF and skin aging remain limited and largely mechanistic. In a seminal proof-of-concept study by our group, SF-rich BSE was topically applied to small skin areas in six healthy volunteers for three days prior to monochromatic UV irradiation [135]. Across UV doses (100–800 mJ/cm^2^ at 311 nm), BSE-treated sites exhibited a mean ~38% reduction in erythema compared with vehicle sites, with inter-individual protection ranging from approximately 8% to 78%. Although erythema is an acute endpoint, it is a validated surrogate marker for UV-induced epidermal and dermal damage that contributes to long-term photoaging.

More recently, we conducted a randomized trial in 18 healthy adults who received 7 days of oral GR, curcumin, or both, following a low-phytochemical diet [223]. Buttock skin was exposed to 2× the minimal erythema dose of UVB before and after supplementation; biopsies were obtained from irradiated and non-irradiated sites. Across all treatment arms, supplementation induced approximately three-fold increases in NQO1 mRNA in skin and reduced transcripts of several pro-inflammatory mediators (IL-1β, TNF-α, IL-6, IL-17, STING, CYR61) in UVB-exposed skin. However, the study did not detect statistically significant changes in clinical erythema or dyskeratotic keratinocyte counts at this sample size and duration, nor did it assess longer-term aging endpoints such as wrinkle depth or elasticity.

Early-phase clinical efforts are also beginning to evaluate SF more directly in skin aging and photodamage settings, although evidence remains limited and definitive results are still lacking. An ongoing early-phase clinical trial from Johns Hopkins (NCT03730649) is evaluating a topical SF formulation applied to both photo-exposed and photo-protected skin sites for up to six months. Planned endpoints include keratin-16/17 expression, UV and visible-light responses, non-invasive elasticity measurements, and clinical aging scores. Results have not yet been reported, but this trial represents one of the first efforts to evaluate SF specifically for skin aging rather than acute photodamage.

Most recently, an open-label single-arm clinical study investigated an oral GR-rich *Brassica oleracea* seed extract in 50 women with mild-to-moderate photoaging. After 56 days, participants demonstrated significant improvements in several objective skin parameters, including reductions in transepidermal water loss and tape-stripping protein loss, increased dermis density, decreased wrinkle depth, and enhanced firmness and elasticity [228]. These findings provide preliminary human evidence that oral delivery of an SF precursor may beneficially modulate barrier function and dermal structure in aging skin. However, the absence of a control group, the short intervention period, and the lack of direct pharmacokinetic or Nrf2-related readouts limit interpretation and underscore the need for controlled trials.

Taken together, the current literature supports SF as a biologically relevant candidate in skin aging and photoaging, with effects on Nrf2 signaling, oxidative stress, inflammatory pathways, and matrix regulation that are consistent with preservation of skin structure and function. Human evidence is promising, though stronger conclusions about anti-aging efficacy of SF will require larger, and perhaps longer, controlled studies. 

## 4. Routes of Administration, Safety, and Pharmacokinetic Considerations

The pharmacokinetics of SF have been investigated through both topical and systemic routes of administration, including oral delivery. While systemic administration may provide broader biological effects, topical delivery offers the advantage of concentrating SF directly within the skin, allowing for more targeted therapeutic interventions in dermatologic conditions. Continued research into optimized delivery methods and application strategies will be paramount to fully realizing the translational potential of this promising compound.

A major translational limitation is that achievable and therapeutically relevant tissue concentrations in human skin remain incompletely defined, particularly for oral administration. At the same time, topical development must reconcile chemical stability with sufficient penetration beyond the stratum corneum to achieve biologically meaningful exposure in viable skin layers. Standardized dose, route, and exposure–response relationships also remain unresolved, making comparisons across studies difficult and continuing to constrain clinical translation.

### 4.1. Topical Delivery

The bioavailability and efficacy of SF for skin conditions can be influenced by its formulation and delivery method. Topical application of SF is a promising approach to enhance local concentration in the epidermis while minimizing systemic exposure. However, the efficiency of its penetration into the stratum corneum and viable epidermal layers is influenced by several factors, including the formulation used for delivery and the integrity of the skin barrier. Theoretically, SF can be incorporated into creams, gels, and ointments designed for direct application to the skin. Notably, vehicles and excipients themselves can influence barrier function and inflammation, which underscores the need for appropriate vehicle controls in study design. Our studies examining topical agents for SF delivery at the Cullman Chemoprotection Center at Johns Hopkins utilized jojoba oil as a vehicle, which along with moringa seed oil, we judged through many unpublished experiments to be the best of the many oils, emollients, creams, and gels examined. Jojoba oil was ultimately chosen as the topical vehicle for delivery of SF used in the clinical trials cited [212,229,230]. In general, formulations containing more organic components, particularly macromolecules, promote rapid reaction and binding of SF, reducing its availability. Thus, while these vehicles may be appropriate for short-term, controlled dosing of highly purified SF in clinical trials, their suitability for topical commercial products remains unclear.

Topical SF has been successfully applied in several mouse models and in human skin, as described in Section 3. Additionally, our group has reported that UVR-induced skin carcinogenesis was substantially inhibited by topical administration of a BSE containing 1 mmol SF in SKH-1 hairless mice; however, the vehicle (80% acetone in water) was completely impractical for human dosing [222]. In a pachyonychia congenita-associated palmoplantar keratoderma mouse model, topical application of SF restored Nrf2 activity, enhanced expression of glutathione reductase and regeneration of GSH, and ultimately reduced the formation of typical skin lesions associated with this condition [231]. Topical SF offers direct epidermal targeting and avoids extensive first-pass metabolism. At the same time, both trial execution and interpretation are complicated by the need to formulate SF in a way that is not only chemically stable but also capable of delivering biologically relevant amounts beyond the stratum corneum into viable epidermal layers. As a result, local tolerability, apparent efficacy, and effective dose delivery may depend substantially on the formulation and vehicle used rather than on SF alone. Because SF is chemically labile, and most studies provide limited formulation detail, it remains difficult to compare dosing approaches, assess reproducibility across studies, or disentangle formulation effects from the intrinsic activity of SF.

Emerging technologies, such as nanoencapsulation, are being investigated to improve the stability and skin penetration of SF when applied topically [232,233]. These nanoformulation strategies demonstrate that ethosomal/ultra-deformable vesicles, nanogels, and liposomal hydrogels can stabilize SF and deliver it effectively into or across the skin. However, these approaches have primarily been evaluated in cancer [234,235,236] and wound-healing models [237], rather than in classical inflammatory dermatoses such as psoriasis or AD, and their clinical relevance for routine dermatologic use remains to be established.

### 4.2. Systemic/Oral Delivery

Systemic delivery of SF (i.p. or s.c.) has also been used in a few studies described in Section 3. These routes allow interrogation of systemic immune compartments (e.g., spleen and lymph nodes) and their contribution to cutaneous disease. Doses are often in the mg/kg or tens of μmol/kg range, likely exceeding typical human dietary exposure. However, tissue-level SF or conjugate concentrations in skin are not systematically reported, which limits quantitative pharmacokinetic and pharmacodynamic interpretation.

Oral formulations, such as capsules containing extracts from cruciferous vegetables rich in GR/SF, have been widely used in non-skin contexts and are discussed in broader reviews [42,43,57,238,239,240]. Both pharmacokinetic and pharmacodynamic responses have been documented in human clinical studies across a dose range of approximately 1–10 µmol/kg/day for SF and GR. At least one study reported a positive pharmacodynamic effect at a dose near 0.1 µmol/kg/day of SF, while multiple studies found no effect in the 0.1–1 µmol/kg/day range [43]. Early work from our group characterized the pharmacokinetics of a single 200 µmol bolus (~2.9 µmol/kg) of SF in human subjects [241], and similar results have since been reported by other investigators. Bioavailability of both SF and GR when delivered orally has been the subject of much work from our group [40,44,242,243,244,245]. In particular, oral exposure can vary substantially depending on formulation and on the efficiency of conversion of GR to SF. In the context of skin disease, we have shown that UVR-induced skin carcinogenesis was reduced by feeding BSE providing daily doses of 10 μmol of GR in SKH-1 hairless mice [246].

To our knowledge, there are only two studies in which oral SF has been evaluated for its effect on dermal conditions in human volunteers. In one study, in which we collaborated, positive effects on melanoma-associated atypical nevi were observed following 28 days of oral SF treatment, and it was well tolerated at 50, 100, and 200 μmol/day, attaining dose-dependent concentrations in plasma and skin [247]. In a recent clinical study, we reported that seven days of oral delivery of an SF-producing dietary supplement induced expression of the cytoprotective gene NQO1 and decreased expression of pro-inflammatory mediators in skin biopsies. Importantly, SF and its metabolites were measurable in skin biopsies after oral GR, providing the first direct evidence that oral SF precursors can reach human skin at pharmacologically active levels [223]. Nevertheless, the short duration of the study limited its ability to demonstrate a clear clinical endpoint. Although a newly published clinical study using oral GR-rich *Brassica oleracea* seed extract supplementation reported positive effects on skin aging, the uncontrolled study design and lack of mechanistic biomarkers prevent definitive confirmation of SF’s efficacy [228].

### 4.3. Safety Profile

SF is a dietary phytochemical and has demonstrated a favorable safety profile [78,239], including in human studies conducted by our group and others, in which SF or BSE has been administered daily for up to six months without evidence of systemic, clinically significant adverse effects [172,223,242,248,249,250,251,252,253,254,255,256,257,258,259,260,261,262,263,264,265]. Safety data of key human SF/BSE studies with skin relevance are summarized in Table S1, with dose, duration, preparation, and reported adverse events (AEs).

Topically applied SF and BSE products have demonstrated good tolerability in both healthy volunteers and patients (Table S1). Mild, transient erythema or stinging has occasionally been reported, likely associated with the vehicle, but no cases of irritant contact dermatitis or photoallergic reactions have been documented. There has been no evidence of systemic toxicity, even with repetitive applications. Across multiple trials conducted in chemoprevention, metabolic disease, and at-risk melanoma survivor populations, oral SF or BSE administered for up to 12 weeks has generally been well tolerated, with mild gastrointestinal upset as the primary AE and no consistent reports of skin toxicity (Table S1). At the same time, safety should not be framed as fully settled for all dermatologic applications. Dose, route, duration of exposure, and disease context remain important variables, particularly because prolonged or excessive Nrf2 activation has shown context-dependent adverse effects in experimental skin systems [82,214]. Thus, the available safety data are reassuring but do not eliminate the need for careful formulation-specific and indication-specific evaluation in future clinical studies.

## 5. Translational Outlook and Challenges

Preclinical studies provide several important insights: (1) Mechanistic depth, with multiple converging lines of evidence implicating Nrf2, HO-1, NF-κB, JAK/STAT, MAPK, inflammasome pathways, and keratin/barrier programming in SF’s cutaneous actions; (2) Disease-specific activity, as SF ameliorates clinical, histologic, and molecular readouts in AD-like, psoriasiform, radiation-induced skin injury, and aging models, and prevents lesion formation in keratin-based genodermatoses; (3) Multicompartment immune modulation, whereby systemic SF modulates T-cell and B-cell subsets (e.g., Th1 and Th17 cells) in psoriasis models, supporting a possible effect on systemic immune processes with cutaneous relevance.

Despite SF’s promising profile, the translation of preclinical success to clinical efficacy faces challenges, notably due to the complexity, chronicity, and immune-driven nature of many dermatologic conditions under discussion. Most clinical investigations to date have been limited by small sample sizes, short follow-up periods, donor-derived extracts with batch variability, and the absence of standardized clinical endpoints. In addition, the evidence base is uneven across diseases, with the strongest human data currently relating to UV-induced skin injury and photoprotection, whereas evidence in psoriasis, AD, acne, and other chronic dermatoses remains largely preclinical. Key translational challenges include: (1) Standardization of Formulations: Variability in SF content among different preparations can significantly affect clinical outcomes. Establishing standardized formulations with reliable dosing and bioavailability will be essential for therapeutic consistency; (2) Individual Differences: Genetic variability in metabolic pathways and microbiota related to SF may lead to differences in individuals’ responses to treatment. Pharmacogenomic research may therefore help personalize SF-based therapies in selected dermatologic contexts; (3) Long-Term Safety: Although current studies demonstrate a generally favorable safety profile for SF, more extensive long-term investigations are required, particularly for chronic dermatologic use; (4) Pharmacokinetics and Tissue Exposure: Achievable and effective tissue concentrations in human skin remain insufficiently defined, especially for oral administration, and formulation instability further emphasizes the need for continued development of optimized topical delivery strategies. Importantly, excessive or prolonged Nrf2 activation may carry risks. Genetic or sustained pharmacologic Nrf2 activation can elicit hyperkeratosis and ichthyosis-like inflammation [214], emphasizing that Nrf2 signaling is not universally beneficial and highlighting the importance of dose optimization and exposure–response characterization [82].

Taken together, SF has a credible translational rationale in dermatology, although its place in clinical practice remains to be defined. Future progress will depend on stronger controlled human studies, improved formulation and delivery strategies, and clearer identification of the disease contexts in which Nrf2-centered pathway modulation is beneficial and clinically relevant.

## 6. Future Directions

While SF exhibits biologic and translational potential in dermatology, supported by evidence from preclinical studies and innovative formulations, continued research is vital to further elucidate its possible clinical applications. This section outlines key research directions and potential barriers to successful implementation.

### 6.1. Optimization of SF Delivery

One of the key challenges in maximizing the translational utility of SF lies in its delivery and bioavailability [266]. Current formulations may not adequately deliver therapeutic concentrations of SF to affected tissues [267]. Research into novel delivery systems, such as unique solvent combinations, liposomes, nanoemulsions, microneedling patch-associated delivery, myrosinase-associated delivery of GR, and microencapsulation techniques, could enhance the skin penetration and stability of SF. However, these approaches will need to be evaluated not only for improved delivery but also for reproducibility and disease-specific relevance. In addition, exploring the manifold synergistic effects of SF [268] with other topical or systemic therapies could improve therapeutic outcomes. For example, combining SF with established anti-inflammatory agents might enhance efficacy while minimizing side effects, although such strategies remain hypothetical until tested in controlled studies.

### 6.2. Personalized Medicine Approaches

Individual variability in response to SF emphasizes the need for tailored therapeutic strategies. Future research should focus on the genetic and phenotypic characteristics that influence how patients metabolize and respond to SF. Pharmacogenomic studies could help identify responders and non-responders, enabling personalized treatment plans. Additionally, since the gut microbiome can influence drug metabolism and efficacy, understanding the interplay between dietary SF intake, skin and gut microbiota, and inflammatory skin conditions can inform the development of effective therapeutic strategies aimed at restoring microbial balance and may offer insights for personalized interventions. As understanding of the skin microbiome evolves, it will also be important to determine whether SF, especially when topically applied, has meaningful effects on skin microbial composition and whether such effects are beneficial, neutral, or context dependent.

### 6.3. Addressing Regulatory and Commercial Challenges

The development of SF-based therapeutics will also require careful navigation of regulatory and commercialization challenges. Standardization of extracts to ensure consistent SF content and quality will be essential for regulatory approval. Setting stringent quality control measures will be necessary for products intended for medical use. Clinical adoption will depend less on general awareness than on the availability of standardized products, robust efficacy data, indication-specific safety information, and reproducible manufacturing. At the same time, SF-containing products may continue to appear in dietary supplement and skin-care markets before stronger dermatologic evidence is available, which makes rigorous clinical evaluation and careful communication especially important.

Overall, future work should focus on optimized delivery systems, disease-specific efficacy testing, personalized response factors, long-term safety, and stronger regulatory and manufacturing frameworks. By addressing these issues, the field will be better positioned to determine where SF has meaningful clinical value and where its role may remain limited or adjunctive.

## 7. Conclusions

SF represents a mechanistically rich small molecule with substantial preclinical activity in models of several cutaneous disorders and skin injury states. By activating Nrf2 and modulating NF-κB, JAK/STAT, MAPK/AP-1, inflammasome signaling, microbiota, and keratin differentiation pathways, SF exerts multi-level effects on oxidative stress, inflammatory cytokine networks, immune and cellular responses, and epidermal structure in AD-like, psoriasiform, and genetic keratinization models, as well as in radiation- and UV-induced skin injury and aging-related contexts.

From a translational standpoint, the strongest human support is presently in UV-related skin outcomes. Topical broccoli sprout or SF-rich preparations have induced protective responses in human skin and reduced UV erythema, with emerging signals in pigmentation and photodamage. By contrast, evidence in chronic inflammatory dermatoses such as psoriasis and AD remains largely preclinical, and acne- or rosacea- related evidence is limited and indirect. Well-designed clinical trials demonstrating therapeutic efficacy are still lacking for several of these chronic dermatologic conditions. Successful translation of SF from bench to bedside will therefore require careful optimization of dose, route of administration, treatment duration, tissue exposure, and formulation, accompanied by rigorous monitoring of clinical efficacy, barrier effects, and safety. Particular caution is warranted because Nrf2 activation is not uniformly beneficial in skin, and excessive or prolonged pathway activation may have adverse effects in some contexts.

Although the evidence base remains predominantly preclinical and important translational questions are still unresolved, the available data support SF as a biologically relevant candidate in dermatology. Its clearest current promise lies in settings where oxidative stress and cytoprotective signaling are especially important, particularly UV-related skin outcomes and photoprotection, where human biologic activity and proof-of-concept signals have already been demonstrated. Broader clinical application, particularly in chronic inflammatory dermatoses, will depend on stronger controlled human studies, improved formulation and delivery strategies, and clearer definition of pharmacokinetic and exposure–response relationships in human skin.

## Data Availability

No new data were created or analyzed in this review.

## Supplementary Materials

The following supporting information can be downloaded at: https://www.mdpi.com/article/10.3390/nu18091444/s1, Table S1: Clinical (A) and preclinical (B) studies of sulforaphane (SF)/broccoli sprouts extract (BSE) with skin endpoints or clear relevance to skin conditions.

## Abbreviations

The following abbreviations are used in this manuscript:

AD	atopic dermatitis
AP-1	activating protein-1
ARE	antioxidant response element
ASD	autism spectrum disorders
BSE	broccoli sprout extract
CCL(2,17, 20, 22)	C-C chemokine ligands (2, 17, 20, 22)
CCN1	cellular Communication Network Factor 1
CXCL8	C-X-C Motif Chemokine Ligand 8
COX-2	cyclooxygenase-2
CYR61	cysteine rich 61
DNCB	2,4-dinitrochlorobenzene
EBS	epidermolysis bullosa simplex
ECM	extracellular matrix
ERK	extracellular signal-regulated kinases
GR	glucoraphanin
Gss	glutathione synthetase
HO-1	heme oxygenase 1
IBD	inflammatory bowel disease
IFN-γ	interferon gamma
IgE	immunoglobulin E
IL (-1β, -6, -8, -17, -22, -23)	interleukin (-1 beta, -6, -8, -17, -22, -23)
IMQ	imiquimod
iNOS	inducible nitric oxide synthase
i.p.	intraperitoneal
ITC	isothiocyanate
JAK	Janus kinase
JNK	c-Jun NH2-terminal kinases
K(16, 17)	keratin (16, 17)
Keap1	Kelch-like ECH-associated protein 1
Ki67	marker of proliferation Kiel 67
MAPK	mitogen-activated protein kinase
MMP	matrix metalloproteinases
NF-κB	nuclear factor kappa-light-chain-enhancer of activated B cells
NLRP3	nucleotide-binding domain, leucine-rich-containing family, pyrin domain-containing-3
NQO1	NAD(P)H quinone oxidoreductase 1
Nrf2	nuclear factor erythroid 2–related factor 2
NSAID	non-steroidal anti-inflammatory drugs
PGE2	Prostaglandin E2
PM(2.5)	particulate matter (2.5)
PPK	palmoplantar keratoderma
Prdx1	peroxiredoxin 1
RORgt	retinoid orphan receptor gamma t
ROS	reactive oxygen species
SAMP1	senescence-accelerated mouse prone 1
s.c.	subcutaneous
SCFA	short-chain fatty acids
SF	sulforaphane
STAT	signal transducer and activator of transcription
STING	stimulator of interferon gene
TGF-β	transforming growth factor beta
Th(1, 2, 17, 22)	T helper (1, 2, 17, 22) cells
TNF-α	tumor necrosis factor-alfa
TSLP	thymic stromal lymphopoietin
UV(R, A, B)	ultraviolet (radiation, A, B)
