# Supplementary material for: Sulforaphane in Cutaneous Disorders and Skin Injury: Mechanisms, Evidence, and Clinical Perspectives
Source: Nutrients. 2026 Apr 30;18(9):1444. doi: 10.3390/nu18091444 (PMC13165143; doi:10.3390/nu18091444)
Supplement: Supplementary file 1 [file nutrients-18-01444-s001.zip › nutrients-4257922-supplementary.pdf]

Table S1. Clinical (A) and preclinical (B) studies of sulforaphane (SF)/broccoli sprouts extract (BSE) with skin endpoints or clear relevance to skin conditions.

Species / indication	Route & preparation	Nominal SF (or precursor) dose	Approx. SF exposure (mg/kg/day)	Duration	Safety / AE-relevant observations	Ref.
			**(A) Clinical Studies**			
**Human – healthy volunteers (Phase I, systemic safety)**	**Oral** BSE: GR-rich (cohorts A,B) or SF-rich (cohort C), q8h	Cohort C: 25 µmol SF q8h = 75 µmol/day ≈ 13.3 mg/day SF<br>Cohort A: 25 µmol GR<br>Cohort B: 100 µmol GR	≈ 0.19 mg/kg/day (70 kg adult)	7 days dosing + 3 days follow-up	Careful clinical and lab monitoring (incl. liver/thyroid): **no significant or consistent subjective/objective toxicities** with any sprout extract ingestion.	Shapiro, Fahey et al. 2006 [1]
**Human – UVB erythema protection (photoprotection, healthy adults)**	**Topical** SF-rich BSE vs vehicle on 2-cm diameter back sites	100-600 nmol SF/site/day ≈ 0.02-0.11 mg/site/day	Local, not systemic	Acute (single/few applications before UVB)	Reduced UVB erythema; **no local irritation or systemic AEs reported**; treated skin clinically normal apart from erythema protection.	Talalay, Fahey et al. 2007 [2]
**Human – keratin-based disorders feasibility (parents of EBS children)**	**Topical** BSE in jojoba oil vs vehicle, inner arm, occluded overnight	500 nmol SF/mL; 1 mL per 3-cm circle ≈ 0.5 µmol/site/day (≈0.09 mg/site/day)	Local, not systemic	7 consecutive days	"Both BSE- and vehicle-treated skin sites appeared normal on clinical and histologic analysis"; "all subjects denied any adverse effects."	Kerns, Guss et al. 2017 [3]

**Human – post-acne macular scars (cosmetic use)**	**Topical** broccoli stem extract cream (contains SF among other phytochemicals) to face	Not quantified in µmol; cosmetic-strength BID to facial scars	Local, not systemic	8 weeks	Significant improvement in erythema/melanin indices; **no adverse skin reactions**; cream described as "well tolerated"; no AE-related withdrawals.	Syahputri, Putra et al. 2025 [4]
**Human – melanoma survivors with atypical nevi**	**Oral** BSE containing SF	50, 100, 200 µmol/day SF ≈ 8.9, 17.7, 35.5 mg/day	≈ 0.13, 0.25, 0.51 mg/kg/day (70 kg)	28 days	All 17 patients completed 28 days with **no dose-limiting toxicities**; no skin-specific toxicity; only mild, nonspecific complaints (occasional GI upset).	Tahata, Singh et al. 2018 [5]
**Human – oral GR→SF ± curcumin, healthy volunteers (skin biopsies)**	**Oral** Crucera-SGS (glucoraphanin) ± curcumin capsules	High-dose GR (450 mg) ≈100–200 µmol SF-equivalent/day ≈17.7–35.5 mg/day	≈ 0.25–0.51 mg/kg/day (70 kg)	7 days	Modulated Nrf2-regulated enzymes in skin; **no serious AEs**; AEs mostly mild GI (bloating, gas, discomfort); no dermatologic AEs.	Chien, Liu et al. 2025 [6]
**Human –Phase II RCT (airborne pollutant detoxification)**	**Oral** beverage with GR + SF	600 µmol GR + 40 µmol SF/day ≈ 24.8 mg/day SF	≈ 0.35 mg/kg/day (70 kg)	12 weeks	**Well tolerated**, no serious AEs; main AEs mild, transient GI symptoms (e.g., gas, loose stools); no signal for skin toxicity.	Egner, Chen et al. 2014 [7]

**Human – GR-rich vs SF-rich crossover trial**	**Oral** GR-rich and SF-rich BSE beverages	800 µmol/day GR or 150 µmol/day SF ≈ 26.6 mg/day SF	≈ 0.38 mg/kg/day (70 kg)	Controlled feeding periods (7 days × 2) with in-between washout (5 days)	Good overall tolerability; no major toxicity signal; no skin-specific issues noted.	Kensler, Ng et al. 2012 [8]
**Human – current smokers (Avmacol® BSSE, detoxification)**	**Oral** broccoli seed & sprout extract tablets	Single-day high doses delivering SF in the **tens of µmol** range (~5–10 mg SF/day)	≈ 0.07–0.14 mg/kg/day (70 kg)	Two weeks × 2 with in-between washout (2 weks)	No CTCAE grade ≥3 events; **most AEs mild** (GI upset, headache); no dermatologic safety signal.	Bauman, Hsu et al. 2022 [9]
**Human – prediabetes RCT (metabolic, systemic tolerability)**	**Oral** BSE containing SF	150 µmol/day SF ≈ 26.6 mg/day SF	≈ 0.38 mg/kg/day (70 kg)	12 weeks	GI side effects (nausea, mild diarrhea, abdominal discomfort) commonest; **no severe AEs**; no reported cutaneous toxicity.	Dwibedi, Axelsson et al. 2025 [10]
			**(B) Preclinical Studies**			
**Mouse – DNCB-induced atopic dermatitis (AD)**	**i.p.** SF (solution for intraperitoneal injection)	2.5, 5, 10 mg/kg/injection, 3×/week	Dose per injection: 2.5–10 mg/kg/day on dosing days; averaged over week ≈1.1–4.3 mg/kg/day	3 weeks (9 injections, concurrent with DNCB)	Body-weight curves overlapped AD controls; **no SF-related mortality or reported organ toxicity**; focus on improved dermatitis and molecular markers.	Wu, Peng et al. 2019 [11]

**Mouse – DNCB-induced AD (second study)**	**s.c.** SF	1 mg/kg/injection, 3×/week	Dose per injection: 1 mg/kg/day on dosing days; averaged over week ≈ 0.43 mg/kg/day	3 weeks (9 injections, after induction of AD)	Improved lesions and cytokine/apoptosis markers; **no mention of death, weight loss, or systemic toxicity**; no dedicated toxicity section.	Alyoussef 2022 [12]
**Mouse – IMQ-induced psoriasis-like dermatitis**	**i.p.** SF	5 mg/kg/day	5 mg/kg/day	7 days (concurrent with IMQ)	SF improved PASI-like scores, histology, and signaling; **no report of SF-related mortality or overt clinical toxicity**; spleen index not worsened vs IMQ alone.	Ma, Gu et al. 2023 [13]
**Mouse – IMQ-induced psoriasis-like dermatitis**	**i.p.** SF	55.3 or 110.6 µmol/kg/day ≈ 9.8 or 19.6 mg/kg/day	9.8 or 19.6 mg/kg/day	14 days total (7 days pre-IMQ + 7 days during IMQ)	Marked improvement in psoriasis-like lesions and Th1/Th17 modulation; **no reported SF-specific toxicity**; mortality not an endpoint in this arm and none noted.	Du, Zhang et al. 2022 [14]
**Mouse – MRL/lpr SLE (systemic autoimmunity with skin involvement)**	**i.p.** SF	82.9 µmol/kg/day ≈ 14.7 mg/kg/day	14.7 mg/kg/day	27 days (starting at 14 weeks of age)	SF **prolonged survival** and improved renal pathology vs vehicle; **no new toxicity signals reported** (no organ damage attributable to SF).	Du, Zhang et al. 2022 [14]

References

1. Shapiro, T.A.; Fahey, J.W.; Dinkova-Kostova, A.T.; Holtzclaw, W.D.; Stephenson, K.K.; Wade, K.L.; Ye, L.; Talalay, P. Safety, tolerance, and metabolism of broccoli sprout glucosinolates and isothiocyanates: a clinical phase I study. *Nutr Cancer* **2006**, *55*, 53-62, doi:10.1207/s15327914nc5501_7.
2. Talalay, P.; Fahey, J.W.; Healy, Z.R.; Wehage, S.L.; Benedict, A.L.; Min, C.; Dinkova-Kostova, A.T. Sulforaphane mobilizes cellular defenses that protect skin against damage by UV radiation. *Proc Natl Acad Sci U S A* **2007**, *104*, 17500-17505, doi:10.1073/pnas.0708710104.
3. Kerns, M.L.; Guss, L.; Fahey, J.; Cohen, B.; Hakim, J.M.; Sung, S.; Lu, R.G.; Coulombe, P.A. Randomized, split-body, single-blinded clinical trial of topical broccoli sprout extract: Assessing the feasibility of its use in keratin-based disorders. *J Am Acad Dermatol* **2017**, *76*, 449-453.e441, doi:10.1016/j.jaad.2016.10.009.
4. Syahputri, F.; Putra, I.B.; Jusuf, N.K. The effect of broccoli stem extract cream (Brassica oleracea L.) on macular scars post-acne. *Frontiers in Medicine* **2025**, *12*, 1680933, doi:10.3389/fmed.2025.1680933.
5. Tahata, S.; Singh, S.V.; Lin, Y.; Hahm, E.R.; Beumer, J.H.; Christner, S.M.; Rao, U.N.; Sander, C.; Tarhini, A.A.; Tawbi, H.; et al. Evaluation of Biodistribution of Sulforaphane after Administration of Oral Broccoli Sprout Extract in Melanoma Patients with Multiple Atypical Nevi. *Cancer Prev Res (Phila)* **2018**, *11*, 429-438, doi:10.1158/1940-6207.Capr-17-0268.
6. Chien, A.L.; Liu, H.; Rachidi, S.; Feig, J.L.; Wang, R.; Wade, K.L.; Stephenson, K.K.; Kecici, A.S.; Fahey, J.W.; Kang, S. Oral Glucoraphanin and Curcumin Supplements Modulate Key Cytoprotective Enzymes in the Skin of Healthy Human Subjects: A Randomized Trial. *Metabolites* **2025**, *15*, 360, doi:10.3390/metabo15060360.
7. Egner, P.A.; Chen, J.G.; Zarth, A.T.; Ng, D.K.; Wang, J.B.; Kensler, K.H.; Jacobson, L.P.; Muñoz, A.; Johnson, J.L.; Groopman, J.D.; et al. Rapid and sustainable detoxication of airborne pollutants by broccoli sprout beverage: results of a randomized clinical trial in China. *Cancer Prev Res (Phila)* **2014**, *7*, 813-823, doi:10.1158/1940-6207.Capr-14-0103.
8. Kensler, T.W.; Ng, D.; Carmella, S.G.; Chen, M.; Jacobson, L.P.; Muñoz, A.; Egner, P.A.; Chen, J.G.; Qian, G.S.; Chen, T.Y.; et al. Modulation of the metabolism of airborne pollutants by glucoraphanin-rich and sulforaphane-rich broccoli sprout beverages in Qidong, China. *Carcinogenesis* **2012**, *33*, 101-107, doi:10.1093/carcin/bgr229.
9. Bauman, J.E.; Hsu, C.H.; Centuori, S.; Guillen-Rodriguez, J.; Garland, L.L.; Ho, E.; Padi, M.; Bageerathan, V.; Bengtson, L.; Wojtowicz, M.; et al. Randomized Crossover Trial Evaluating Detoxification of Tobacco Carcinogens by Broccoli Seed and Sprout Extract in Current Smokers. *Cancers (Basel)* **2022**, *14*, 2129, doi:10.3390/cancers14092129.
10. Dwibedi, C.; Axelsson, A.S.; Abrahamsson, B.; Fahey, J.W.; Asplund, O.; Hansson, O.; Ahlqvist, E.; Tremaroli, V.; Bäckhed, F.; Rosengren, A.H. Effect of broccoli sprout extract and baseline gut microbiota on fasting blood glucose in prediabetes: a randomized, placebo-controlled trial. *Nature Microbiology* **2025**, *10*, 681-693, doi:10.1038/s41564-025-01932-w.

11. Wu, W.; Peng, G.; Yang, F.; Zhang, Y.; Mu, Z.; Han, X. Sulforaphane has a therapeutic effect in an atopic dermatitis murine model and activates the Nrf2/HO-1 axis. *Mol Med Rep* **2019**, *20*, 1761-1771, doi:10.3892/mmr.2019.10405.
12. Alyoussef, A. Attenuation of experimentally induced atopic dermatitis in mice by sulforaphane: effect on inflammation and apoptosis. *Toxicol Mech Methods* **2022**, *32*, 224-232, doi:10.1080/15376516.2021.1994076.
13. Ma, C.; Gu, C.; Lian, P.; Wazir, J.; Lu, R.; Ruan, B.; Wei, L.; Li, L.; Pu, W.; Peng, Z.; et al. Sulforaphane alleviates psoriasis by enhancing antioxidant defense through KEAP1-NRF2 Pathway activation and attenuating inflammatory signaling. *Cell Death Dis* **2023**, *14*, 768, doi:10.1038/s41419-023-06234-9.
14. Du, P.; Zhang, W.; Cui, H.; He, W.; Lu, S.; Jia, S.; Zhao, M. Sulforaphane Ameliorates the Severity of Psoriasis and SLE by Modulating Effector Cells and Reducing Oxidative Stress. *Front Pharmacol* **2022**, *13*, 805508, doi:10.3389/fphar.2022.805508.
